# Eleven quick tips for data cleaning and feature engineering

**DOI:** 10.1371/journal.pcbi.1010718

**Published:** 2022-12-15

**Authors:** Davide Chicco, Luca Oneto, Erica Tavazzi

**Affiliations:** 1 Institute of Health Policy Management and Evaluation, University of Toronto, Toronto, Ontario, Canada; 2 Dipartimento di Informatica Bioingegneria Robotica e Ingegneria dei Sistemi, Università di Genova, Genoa, Italy; 3 ZenaByte S.r.l., Genoa, Italy; 4 Dipartimento di Ingegneria dell’Informazione, Università di Padova, Padua, Italy; McGill University, CANADA

## Abstract

Applying computational statistics or machine learning methods to data is a key component of many scientific studies, in any field, but alone might not be sufficient to generate robust and reliable outcomes and results. Before applying any discovery method, preprocessing steps are necessary to prepare the data to the computational analysis. In this framework, data cleaning and feature engineering are key pillars of any scientific study involving data analysis and that should be adequately designed and performed since the first phases of the project. We call “feature” a variable describing a particular trait of a person or an observation, recorded usually as a column in a dataset. Even if pivotal, these data cleaning and feature engineering steps sometimes are done poorly or inefficiently, especially by beginners and unexperienced researchers. For this reason, we propose here our quick tips for data cleaning and feature engineering on how to carry out these important preprocessing steps correctly avoiding common mistakes and pitfalls. Although we designed these guidelines with bioinformatics and health informatics scenarios in mind, we believe they can more in general be applied to any scientific area. We therefore target these guidelines to any researcher or practitioners wanting to perform data cleaning or feature engineering. We believe our simple recommendations can help researchers and scholars perform better computational analyses that can lead, in turn, to more solid outcomes and more reliable discoveries.

This is a *PLOS Computational Biology* Software paper.

## Introduction

With the huge spread of applied machine learning and data science, researchers have employed computational tools to analyze data of different types worldwide, for multiple scientific and industrial goals. However, not all the data science studies succeed. As Pedro Domingos clearly described [1]: “At the end of the day, some machine learning projects succeed and some fail. What makes the difference? Easily the most important factor is the features used” [1]. And he is completely right: as surprising as it might sound, the key factor of success in computational analyses is not the machine learning or statistic method used, but rather how the data are processed before the analytics phase.

Specifically, data cleaning (or data cleansing), the procedure of removing or handling the nonstandard elements from a dataset, such as outliers, duplicates, null data instances, or similar [2–5]. Feature engineering is the process of creating new variables (usually encoded as columns in a dataset represented as a table) from the existing ones, by merging or processing 2 or more existing features-columns into a new more informative one [6,7]. Of course, preprocessing the data before the analytics phase is alone not sufficient to generate valid scientific results. More importantly, the data should be representative and informative, the experiments should be well designed, and the scientific question should be well posed. If one or more of these aspects is missing, no meaningful results can be obtained by the scientific analysis, even in presence of a well-performed preprocessing phase. In any case, data cleaning and feature engineering, even if very important, are sometimes underestimated or forgotten by data science beginners and students.

The PLOS Computational Biology Education collection provides quick tips for initial data analysis [8], for network data analysis [9], and for biocuration [10], but not for data cleaning and feature engineering. An interesting study by Jan Van den Broeck and colleagues [3] presented some general guidelines for medical data cleaning regarding data collection, but unrelated to computational analyses. In this article, we provide quick tips on data cleaning and feature engineering to be followed by any data scientist working in any scientific field, to avoid common mistakes and misleading results.

Before proceeding in more detail with a look at these concepts, it is useful to point out why these precautions are so important: The lack of data cleaning and feature engineering steps in a computational analysis can lead to deceiving results that, if not caught on time, might have a dangerous impact on the subjects of a scientific research study. Practitioners working on computational biology or health informatics and handling data of patients should pay particular attention to the recommendations we make here. Problems generated by noise or corrupted data are particularly evil, since they hide themselves in datasets that look normal. Applying these tips can allow researchers to detect and handle these sometimes invisible problems.

## Tip 1: Keep things simple: Know your data and methods

The general principle that we recall in this tip is to keep things as simple as possible. This principle, often referred as Occam’s Razor principle [11], means that one always has to search for simple solutions since simplicity is a goal per se, but it does not mean that simplicity always lead to better performance [12]. In this context, “search” is a necessary word since, as ensured by the No Free Lunch Theorem, there is no way of selecting a method prior to testing it on the data [13,14]. For example, before searching for nonlinear correlation between variables, one starts with the linear one (Tip 3) or when one wants to map a free text, it uses first bag of words and then a pretrained network (Tip 8). Consequently, in data analysis, the simplicity of the solution depends on two main factors: the methods and the data. With regards to methods, it has to be considered that, in most applications, complex approaches, complex methods lead to improvements that are practically negligible (namely improvement of a few percentage points of accuracy) [15–17], at the cost of a huge increase in complexity (namely difficulty in fully understanding the method, decrease in interpretability of the results of the method, increase in its implementation complexity, and increase in its computational requirements) [18–20].

For example, in missing data imputation, one starts replacing the missing value with mean/mode before using nonnegative matrix factorization (Tip 4). Nevertheless, in some cases (Tip 8), using complex methods is necessary to achieve satisfying performances [21–23]. In fact, complexity is always a relative concept: Using deep learning over shallow learning (for example, for handling graph, sequences, or text data) could be necessary, but within the deep learning framework there are complex state-of-the-art methods whose performance can be almost matched with many basic approaches [24,25].

Finally, one cannot simply look at the complexity of the methods alone, but must also look at the complexity of the solutions: Most of the time, even very complex methods use simple solutions [26–28]. For example, one can see how much difference there is in missing data imputation between replacing mean/mode and using nonnegative matrix factorization. When it comes to data, it is often more important to have high-quality data with respect to using very complex approaches [29,30]. In fact, the bad quality data increases the possibility of false discovery (garbage in garbage out), while good quality data can lead to strong increase in performance of simple methods [31–33]. Tip 2 deals exactly with this issue: When one collects data, a way to improve their quality is to remove the technology-induced data.

Data cleaning and feature engineering exactly address this problem [34–36]: If one cannot improve the data by performing again or increase in cardinality/quality the data collection procedure (for example, because one has to use existing data or collecting more data takes years), it is at least required to put the data in the best shape for the analysis (Tips 2 to 5).

Finally, it is fundamental to know well the used methods (namely working hypothesis, strong and weak points, and their limitations), coupling it with statistically sound and funded methods, which allow to avoid trivial errors (for example, false discovery and data snooping) [37–39]. For example, when one uses the correlation coefficient just linear correlation can be found, while nonlinear correlation may still exist. When, instead, one uses principal component analysis for dimensionality reduction, they have to remember that it works well on data coming from a multivariate Gaussian distribution (Tip 3). In fact, even for top researchers in data analysis, preventing false discovery is very hard since errors may be hidden both in caveats or in commonly used procedures [40–43].

When analyzing data of electronic health records (EHRs) of patients with myocardial infarction to predict their readmission and mortality, for example, remarkable results often can be obtained through simple conventional statistics models, without employing machine learning methods [44]. In a nutshell, simplicity based on the structure of the data is often the key to successful method selection.

## Tip 2: Take care of the technology-related noise

Some datasets contain data that were collected without sophisticated biomedical engineering technologies, and therefore their data are ready to use immediately. EHRs that include administrative data such as sex, age, weight, height, history of smoking, or history of diseases, for example, usually contain a reduced, non-systematic amount of particular noise or errors introduced during the data collection phase, and can therefore be used as they are.

Other datasets, on the opposite, are composed of data gathered through advanced biomedical engineering machines, which might have introduced noise during the data collection. This is clearly the case, for instance, of batch effect for microarray and RNA-Seq gene expression datasets [45,46]. Batch effects happen because different conditions and/or different reagents or substances are employed in different moments to collect data that are part of the same dataset.

It is well known and studied that these differences can actually impact on the quality of the final gene expression data. To solve this issue, researchers introduced several techniques for batch correction [47–51]. A similar issue happens for electrocardiogram (ECG) data that often suffer from noise introduced by the signal-recording machines [52] and for medical images, where the problem can be tacked through denoising techniques [53–55].

Our suggestion is not to take the dataset blindly as it is, but to rather investigate if its data type has some typical noise to handle. Computational analyses done without considering the noise within the data can lead to misleading results and, in the best case, need to be completely redone after the discovery of this issue, which can happen during the journal review.

## Tip 3: Gain confidence with the data: Perform an exploratory data analysis (EDA)

This apparently simple tip suggests to become familiar with the data (for example, by understanding the data types and by visualizing and summarizing them) in order to reduce the workload during more advanced analysis, as explained further in the next tips. This step is commonly referred as exploratory data analysis (EDA) [56].

Nevertheless, EDA is not always simple, since it is a time-consuming step that requires a combination technical skills (knowing many software libraries), experience (which approach to use), and domain knowledge (some data may need some preliminary corrections, as explained in Tip 2) [57]. As first step, it is necessary to retrieve, to understand, and, if needed, to complete the metadata (data that are necessary to understand the actual data) possibly with a tight collaboration with a domain expert or with the people who produced the data. This will allow also to spot from the beginning potential confounding factors [58]. Then, we can start with the actual EDA.

Let us start with tabular data, namely, data that can be fully represented with one or more tables where each column is a feature and each row is a sample. In health informatics, often data of EHRs are represented as tables with patients on the rows and clinical features on the columns [44,59]. The first step of EDA is to understand what type of data it is: quantitative (continuous or discrete) and qualitative (nominal or ordinal). Afterwards, one can summarize the data with simple descriptive statistics (for example, mean, median, mode, quantile, symmetry, and kurtosis), visualize the data with simple or interactive visualizations (for example, bar plots, pie charts, scatterplots, or lines), and combine the two approaches (for example, visualizing the distribution of the data with a line plot or an histogram).

One can also easily search for relations between variables using the Pearson product-moment correlation coefficient (PCC), which tells if two variables are linearly correlated, or use pairwise scatter plots to achieve the same goal also detecting nonlinear correlations. These procedures can and should be made as automated as possible by fully exploiting the metadata (for example, to analyze columns bases on the data type they contain). For this purpose, there are plenty of free software libraries in R [60] and Python [61], such as ggplot2 [62], matplotlib [63], and plotly [64]. There are also some nice high-level GUI-based software programs for this purpose like KNIME [65] or Tableau [66]. After these initial inspections, more sophisticated EDA can be performed on the available tabular data. A first possible analysis involves the usage of clustering techniques that allow to group data based on some notion of similarity [67]: this notion might be explicit for linear techniques (for example, Euclidean or Manhattan distance) or implicit for nonlinear ones.

Many techniques [68] and related software libraries [69] exist for clustering tabular data, from simple methods (for example, k-Means, Affinity, Propagation, Mean-Shift) that basically require the data groups to be linearly separable to more complex ones (for example, Spectral Clustering, Agglomerative Clustering, DBSCAN) that relaxes the hypothesis of linearity. A second important analysis concerns the effort to reduce the dimensionality of the data [70,71] that allows for better human understanding (or better visualization) and to alleviate the “curse of dimensionality” (namely the increase in complexity of extracting meaningful information as the number of features grows) [72].

Moreover, in this case, many techniques [73] and software libraries [74,75] exist: from simple methods (for example, Principal Component Analysis, Singular Value Decomposition, and Linear Discriminant Analysis) to more complex ones (for example, Isomap, Self Organizing Maps, and t-SNE) that again try to model data which lie in a linear or a nonlinear subspace of the original feature representation, respectively. We explain more in detail how to handle tabular data in Tip 7. Regarding non-tabular data, such as structured data such as graphs or natural text, or sequences/time series, the EDA becomes more complex since data are characterized by entities that can be represented, for example, with tabular data and the relations between them. For this kind of data, dedicated software packages exist for exploration and visualization [76–78]. To make more advanced EDA on non-tabular data (for example, with clustering or dimensionality reduction), it is necessary first to map them into tabular data; this aspect is the focus of Tip 8.

Thousands of biomedical articles include an EDA; among them, a good example of EDA is the recent study by Dey and colleagues [79] about the epidemiological outbreak of Coronavirus Disease 2019 (COVID-19). At the end of this phase, the analyst should have a clear knowledge of the metadata related to the available dataset, the most important descriptive statistics related to the data, the relations between the available features, and an idea of how samples are distributed in the feature space.

## Tip 4: Look for missing data and handle them properly

When working with real data produced in the biomedical contexts, missing data is one of the most common issues encountered in the cleaning process. Each of these occurrences, usually represented by an NA (Not Available or No Answer) or an empty field in the data, corresponds to the absence of a known value for one observation of a variable in the dataset. In the context of healthcare, the failure to detect or record information may be caused to a variety of reasons, such as the need to perform different tests on the patients depending on the evolution of their clinical condition, shortcomings in the insertion phase, specific characteristics of the technologies used to generate the data, and/or administrative requirements.

In some situations, however, a missing value could also indicate (or be related to) a negative response, rather than representing a value that was not collected or recorded. All these cases originate in the data collection and therefore require some knowledge of the acquisition process to be recognized and handled correctly: for example, an “ongoing therapies” field might be left blank if the patient is not taking any medication, if the question was not asked, if the name of the medication was not known to the subject, or if it was known but not recorded. In general, missing data can reduce the representativeness of the sample and consequently introduce possible bias in the inferences about the population, leading to invalid conclusions [80,81].

Therefore, in the descriptive phase of a preprocessing phase, it is recommended to investigate the amount and the patterns of missingness, together with the relationships between missing and non-missing values. Only if the values are missing completely at random (MCAR), that is, there is no relationship between the missing data and other observed or unobserved values within the dataset, can the data sample still be considered representative of the population; in all other cases, when the data are missing at random (MAR) or missing not at random (MNAR), some bias can be introduced in the study of descriptive patterns and causal effects [82]. There are several tools that allow to explore, visualize, and describe missing data, such as the Missingno Python software library [83] or the naniar R software package [84].

Ideally, the best way to deal with missing data is to avoid them altogether by carefully planning the study and data collection; however, in the practice, missing data happens in almost every study. Some analytic tools, including machine learning algorithms, can automatically detect and deal with missing data. However, most of them require complete information. There are several approaches to obtaining a complete dataset.

The simplest way is to apply filtering techniques to exclude all unrecorded information from the analyses by either dropping the cases where at least one variable is missing (complete-cases analysis) or those variables that have at least one missing occurrence (complete-variables analysis). While being extremely simple, filtering could not be a viable option when the sample cardinality is limited and/or the percentage of missing data is too high. Moreover, it has the drawback of ignoring possible relationships between variables, which could cause information loss and introduce bias [85]. More sophisticated approaches allow missing values to be replaced (or “imputed”) with plausible values inferred from available data. Simple statistical approaches, such as filling the missing values with the mean, median, or mode of the observed values, or by propagating dynamic values (for example, Last Observation Carried Backward or Next Observation Carried Forward), are often applied. These methods are fast and easily interpretable, but may lead to low accuracy and biased estimates of the investigated associations [81,86]. Alternatively, more sophisticated model-based imputation techniques can be used: with these approaches, a predictive model—based, for instance, on regression techniques [87,88], Artificial Neural Networks [89,90], or k-Nearest Neighbors [91,92]—is created to estimate values that will replace missing data [93,94].

Remarkably, some of these approaches are also able to manage cross-sectional time series data by exploiting their dynamic nature for estimating the imputed values [95–98], and to deal with the different types of variables that can constitute clinical datasets [87,92]. Furthermore, different estimates of the value to be replaced can be used in multiple imputation strategies in order to limit possible noise due to estimation uncertainty [99]. Ultimately, the best strategy for handling missing data must be assessed on a case-by-case basis, taking into account a number of factors, including the cardinality of the missing data with respect to the available data or specific assumptions about their distribution/relationships, always bearing in mind that improper handling of missing data might lead to inconsistent analysis.

Moreover, when it is unclear whether and how missing values could influence the outcomes, sensitivity analyses can be performed to assess the impact of different imputation/management strategies of the missing information in the specific case study [100]. In all cases, we recommend accurately reporting what the data originally looked like and what was done for the sake of transparency and reproducibility.

## Tip 5: Look for inconsistencies, duplicates, outliers and handle them properly

Another aspect to consider during a data cleaning phase is the detection of inconsistent data, duplicate date, and outliers. For inconsistencies, we mean feature data whose values do not comply with the expected range, format, or value: for example, negative ages of patients, dates where the day number is greater than or equal to 32 or the month is greater than or equal to 13, negative salary, and similar. These inconsistencies regard only one single feature and are called pointwise.

Of course, it is easier to spot them when they are related to known common variables (such as age, dates, etc.), and more difficult to detect when related to biomedical factors with which a researcher might not be familiar. Inconsistencies can also be related to multiple variables, if their values are correlated to several factors. For example, a patient might have a year indicating their death in the “death date” variable and then result alive in the “current condition” feature: it is clear that one of the two values is wrong, even if it is impossible to say which one immediately.

Inconsistencies can be detected by computing the ranges of each feature of the dataset and analyzing them carefully in respect to the dataset documentation. Differently from inconsistencies, duplicates are pairs of features whose values have identical meanings, even if they might appear with different names. For example, a “sex” variable where 0 indicates men and 1 indicates women and another factor named “gender” containing the string values “male” and “female” might be duplicate features. Duplicates can be detected by comparing each possible pair of variables of a dataset: if found, we recommend to remove the least informative one of each couple. An outlier is a data point whose value is inconsistent with the rest of the dataset. It represents an anomaly or an error, and of course, it is impossible to say if its value makes sense or not beforehand: An analysis of the outliers can be done only when the correct reasonable range of values of each feature is known. In any case, it is important to detect outliers and try to understand if they should be removed or not.

As a rule of thumb, we suggest to flag all the values of a feature that are at least ten times the median value (except when dealing with exponentially distributed data). Other techniques involve the visualization of boxplots or the usage of more sophisticated methods such as the Hidiroglou–Berthelot method [101], Rosner’s test [102], and Dixon’s test [103]. Unidimensional analysis outliers regard the distribution of values within a single variable. Outliers, however, can regard several variables at the same time. In these cases, novelty and outlier detection methods for multidimensional analysis [104] or unsupervised machine learning algorithms [105] can be employed.

## Tip 6: Prepare the data for your algorithm

An important element to bear in mind, when one has to perform data cleaning and feature engineering, is that this step is seldom independent from the algorithms to apply in the later analysis [106,107]. This means that data preparation is algorithm dependent, hence not always the procedures previously described (for example, in Tips 3 to 5) are necessary at all or needed to be performed in the same way.

Let us describe a few cases to better understand our statement: When one uses particular novel state-of-the-art models, there may be no need for dimensionality reduction [108] or, when using robust losses in neural networks, outlier removal can be avoided [109]. In the scientific literature, there are two main families of algorithms to perform data analysis: shallow and deep [19,110]. Shallow models need the data to be already arranged in a specific representation before the learning phase; deep models, instead, learn the representation space directly from the data. Shallow models are usually the best option for tabular data where deep models do not give noticeable advantages (Tip 1), but a deep model can be an asset when employed in structured data (images, graphs, sequences, and natural language) and when shallow models cannot compete nor achieve satisfying performances. In this tip, we focus on tabular data, while Tip 8 is about structured data and how to map them into tabular data. In order to prepare the data for an algorithm, one needs to check four main aspects. The first one is the ability of the algorithm to handle non-quantitative variables (for example, nominal or ordinal): some algorithms can (for example, rule-based methods) while some others cannot (for example, kernel methods).

If not, one has to transform these variables into quantitative variables with one of the different techniques available (for example, the one-hot coding for the simple ones) [106,111,112]. The second check is to understand the effect of normalization on the performance of the algorithm (for example, if all features need to have a similar range). Some algorithms (for example, Decision Trees and Random Forests) need no normalization but other algorithms (for example, Artificial Neural Networks [113]) are deeply influenced by the normalization approach (for example, min/max or Pareto scaling) that need to be carefully selected [106,114,115]. Another important check to perform on the algorithms is their ability to handle large numbers of variables (features), or when they start to suffer from the curse of dimensionality (namely when their performances start to decrease, increasing the number of features).

Some algorithms suffer less from this problem (for example, modern overparameterized method [108]), but other do (for example, Random Forests) and in this case, we have to reduce the dimensionality of the data (for example, with the dimensionality reduction approaches described in Tip 3 or with wrapper methods, like the ones based on the permutation test, which actually reduce the feature space based on the target analysis) [116–119] taking into account important problems like collinearity or confounding [58,120]. When the analyzed datasets have a huge number of variables, feature selection methods can be used to scale down the feature space [121]. Finally, the algorithms always have a limit in the number of samples that they are able to handle based on their computational complexity.

Usually, shallow methods (for example, Kernel Support Vector Machines) have limitations when dealing with some tens of thousands samples [59], while deep methods (for example, Graph Neural Networks) can handle million samples [19,110]. When the available dataset is too big for our algorithm, we have to subsample it, and many techniques exist for this scope (for example, simple random subsampling) [122–124]. For example, a study by Ahmed and colleagues [125] employed a class-imbalanced subsampling lasso technique for inferring adverse drug reactions. At the end of these phases, the data analyst should have clear in mind the type of data preparation needed for the following analysis if they know their methods (Tip 1) and their data (Tips 2 to 5).

## Tip 7: Appropriately feature engineer tabular data

Regarding feature engineering on tabular data, there are different approaches that can be also combined together [6,7,35,36]: domain-specific feature engineering, explicit or implicit feature mapping, and learned feature mapping. Domain-specific feature engineering refers to the process of generating new variables from the ones already present in the tabular representation, which are known (based on experience and scientific domain knowledge) to be predictive for the specific problem at hand. This step is fundamental since it allows us to encapsulate all the prior scientific knowledge of the domain experts in the data and will simplify the subsequent analysis by allowing the use of simpler models (Tip 1) and will allow to achieve better performance [126–128].

The limitation of this approach is the amount of scientific domain knowledge available, easily retrievable, or synthesizable in new features [129,130]. Explicit or implicit feature mapping deals with the problem of building nonlinear models in multidimensional spaces. In fact, even after a careful domain-specific feature engineering phase, a simple linear model might be insufficient to achieve good performance.

For this reason, it may be useful to map the data into a higher dimensional space and then apply a linear model, which is equivalent to building a nonlinear model in the original space [110]. This mapping might be explicit [7] (for example, inserting all the possible linear and nonlinear combinations of the features) or implicit [131,132] (for example, using kernel methods). In the first case, the approach will allow the use of more interpretable models [18], but it could easily stumble into the “curse of dimensionality” [133], and this is why implicit methods are more popular even though less interpretable. The limitation of this approach is that usually the number of generated features is huge with respect to the necessary ones [19,110]. Finally, learned feature mapping is the most advanced and powerful method [19,134]. In fact, domain-specific feature engineering and explicit or implicit feature mapping have the drawbacks of generating a fixed feature mapping not customized for the problem at hand [135].

Learned feature mapping (for example, via deep neural network) allows for learning (namely generating) features customized for the problem at hand, by potentially reducing the number of features to the minimum necessary number. The limitation of this approach is that one is using the same data both to engineer features and to perform the analysis, which usually requires a huge amount of data [136–138]. When it comes to tabular data, domain-specific feature engineering and explicit or implicit feature mapping are usually the most used and effective approaches often exploited simultaneously. Learned feature mapping usually does not give appreciable advantages apart from the case of availability of huge amounts of data. An interesting example of tabular data feature engineering was described in the study of Spänig and colleagues [139] on classification of peptide encodings.

## Tip 8: Appropriately feature engineer non-tabular data

Engineering features on non-tabular data is a key step in data preprocessing [140–142]. With feature engineering for non-tabular data, we refer to feature engineering for images [143,144], graphs [145,146] (with trees [147–149] and sequences/time series [150–153] as particular case), and natural language [154,155]. As for tabular data (Tip 7), feature engineering for non-tabular data involves different approaches to the problem that can be also combined: domain-specific feature engineering, explicit or implicit feature mapping, and finally learned feature mapping.

All these approaches try to map complex structures into tabular data (for example, a graph into a series of features). The difference with feature engineering for tabular data is that, in order to obtain satisfying results for non-tabular data, domain-specific feature engineering and explicit or implicit feature mapping are usually not enough and learned feature mapping needs to be applied simultaneously with the other techniques [19,110].

Domain-specific feature engineering for non-tabular data tries to search for the presence of important substructures or important pieces of information, removing the unimportant ones, in the original data. For example, in image analysis, it is important to remove artifacts and restrict the analysis to particular parts of the image [156], for example, when analyzing the data of The Cancer Imaging Archive (TCIA) [157]. In graphs, trees, and sequences analysis, it is important to search for particular substructures (subportion of a graph) and substructure properties (graph node labels and properties) that are known to be relevant for the analysis [158]. In natural language analysis, it is important to correctly preprocess the text (for example, handle capitalization, tokenize the text, remove stopwords, stemming, and lemmatization) and then search for the presence of particular important words or group of words (n-grams and frequencies) [159].

Obviously, this approach allows for encapsulating all the domain knowledge but it is also time consuming, computationally inefficient, and in some sense oversimplified, namely, we risk to miss or disregard important information. Explicit and implicit feature mapping try to fill the lack of the domain-specific feature engineering by trying to exhaustively coding the non-tabular information into a series of features. For example, in graph explicit feature mapping can be done via Random Walk or Weisfeiler–Lehman (namely considers subtree-walks where nodes are repeated).

In sequences, instead, explicit feature mapping can be done in different ways like splitting the sequence in subsequences (consequential or not and overlapping or not) and then computing properties of this subsequence (for example, mean, mode, median, and variance). Unfortunately, explicit feature mapping has two drawbacks. The first one is that it may become computationally expensive or prohibitive to compute. The second one is that many features will be probability irrelevant for the analysis unleashing the curse of dimensionality. The first issue can be faced by exploiting implicit feature mapping where the prominent approach is the one of the kernel methods that will require the use, in the following analysis, a kernelled method [131]. For example, graph analysis and implicit feature mapping can be done via diffusion kernels. But there are also special kernels for images, sequences, and text [146,147,152].

For the second issue related to explicit and implicit feature mapping, learned feature mapping is the solution. In fact, learned feature mapping (via deep neural network for non-tabular and structured data) allows for learning (namely generating) features customized for the problem at hand potentially reducing the number of features to the minimum necessary number. The difference, with respect to the case of learned feature mapping for tabular data, is that in the case of non-tabular data, the approaches for learned feature mapping are becoming quite effective and efficient thanks to the availability of huge amount of data that allows to pretrain model in one application that can be applied and fine-tuned to other problems [160–162]. These pretrained models map complex data structures into reasonably small cardinality yet quite expressive features that can be reused efficiently and effectively in many applications. For example, when analyzing data of Gene Ontology annotations that are arranged following a directed acyclic graph (DAG), ad hoc methods that exploit this ontology structure can be more effective than more sophisticated computational intelligence techniques [119,163,164]. The take home message of this Tip 8 is that feature engineering for non-tabular data is quite similar to the one for tabular data (Tip 7), except for the fact that it is often necessary to use learned feature mapping.

While learned feature mapping might require a huge amount of data and computational effort to be effective, in the case of structured data, many pretrained networks are freely available in the computer science’s literature, and they can be used to directly map the non-tabular information into tabular one or can be easily fine-tuned with a reasonably small amount of data.

## Tip 9: Make the preprocessing trustworthy

The increasing complexity and amount of data available strongly demands analytics tools able to automatically handle both tabular (Tip 7) and non-tabular (Tip 8) data [165–167]. In fact, more and more often, analytics tools are exploited by domain experts rather than data scientists, and this requires tools that can be easily exploited by the former [168–170].

Hence, data and tools should not only be collected and designed to achieve high technical and functional standards but they should be something that we humans can trust, fulfilling the requirements of fairness, robustness, privacy, and explainability [142,171–175]. With fairness, we intend the ability of the data and tools to not discriminate subgroups in the population based on, for example, gender, race, and political or sexual orientation [176–178].

For this purpose, different quantitative measures of (un)fairness exist to assessing the phenomenon that can be grouped into statistical (namely referring to the subgroup of the population) and individual (namely referring to the single individual of the population) measures [178]. An example of statistical fairness is the difference in the distribution of the feature coding the sex in our data (namely the demographic parity), while an example of individual fairness is what would happen to the representation of a sample if we change just the features correlated to the sex (counterfactual fairness).

Aside from measuring it, many techniques exist to mitigate unfairness, ranging from solutions that act in the preprocessing phase [177] (Tip 6) to the ones that act when learning representations [179] (Tips 7 and 8). For example, a way to make our engineered features fairer with respect to the demographic parity is to impose their distribution in the subgroups of the population as similar as possible (for example, minimizing the Sinkhorn distance [180]).

With robustness, we intend the ability of the tools and results to be resilient to changes (natural or malicious) in the data [142,181–183]. In fact, many algorithms that are currently used on a daily basis to perform data analysis, and especially the ones developed for non-tabular data (Tip 8), are prone to perform poorly when data are carefully, even lightly, modified. For example, it is possible to easily find the minimal modification to a data sample that allows to change the results of the final analysis [181]. This creates problems in real world applications where decisions can have a critical effect on the life of people: In fact, this vulnerability may generate a surface of attack to the data-driven systems to be maliciously induced into mistakes. Nevertheless, this property also creates the possibility of generating synthetic data that well resemble the real data distribution (for example, to enrich our dataset, or to release data without compromising the privacy of the original real data) in a smart way [182–184].

Privacy-preserving data analysis addresses the self-contradictory problem of keeping private information about individual observations while learning useful information about a population [142,185–187]. This is a fundamental problem when one has to release or deal with sensitive data. Before understanding possible mitigation to privacy leaks, it is important to understand the threats. The first one is the possible intentional/unintentional internal/external threat due to keeping and releasing sensitive information, while the second one is its reconstruction, namely, the possibility of reconstructing the raw data starting from the processed ones.

Current solutions have to deal with two main scenarios, namely when data needs to be kept centralized and handled by a trusted curator (anonymization and perturbation techniques), or decentralized eliminating the curator risk (cryptographic or distributed protocol techniques), even if hybrid scenarios have started to appear [188–191]. For example, anonymization techniques (k-Anonymization, l-Diversity, and t-Closeness) try to maintain the privacy of the data subjects by obscuring personally identifying information within a dataset while preserving data utility.

Perturbation techniques (Differential Privacy) instead exploit noise to corrupt the data quantifying the disclosed information in terms of the power of the noise. Cryptographic techniques (Homomorphic Encryption) allow to work on encrypted data as the computations are performed on their original non-encrypted version at the cost of a high computational overhead and the limitations for some operations. Distributed protocol techniques (Secure Multiparty Computation) defines algorithms where different participants, each owning their private data, want to compute some aggregated results while keeping the secrecy of their inputs.

Explainability aims at providing explanations for the data analytics process engendering trust in their users [18,192–194]. In some cases, in fact, the analysis is explainable by design (Tips 2 to 5) while others may be much harder to understand (Tips 7 and 8). Nevertheless, a formal, rigorous, and commonly accepted definition of explainability does not yet exist and is strongly dependent from the application. All the definitions tend to describe explainability as the degree to which a human can understand (for example, with examples, with concepts, or with model approximation) the cause of a decision, or the degree to which a human can consistently predict the analysis’s result (for example, understanding where the model focuses its attention, what features have the highest importance, and what would happen by slightly changing the analysis) [195–198]. To evaluate the interpretability, we have to follow three criteria: the specific application at hand, the skills of the people that will receive and use the explanation, and the functional requirements, namely how the explanation should be made [199,200]. To get interpretable machine learning models, different approaches exist [18,192–194].

First, the methods can be divided into model specific (namely approaches that work only for specific analyses, like Attention Maps for deep neural networks) and model agnostic methods (that are approaches that work only for a group of analyses, like the Local Interpretable Model-Agnostic Explanations method). These two families can be further subdivided based on their ability to provide global (explanation of the entire analysis) or local (explanations of a specific output of the analysis) interpretability.

Additional information and insights about the role of trustworthy machine learning in healthcare and medical sciences can be found in several studies [201–204]. The take-home message here is that, when performing data cleaning and feature engineering, technical and functional standards are not the only metrics to take into account, but one needs to be sure that the data and the tools fulfill the requirements of fairness, robustness, privacy, and explainability since each scientific study will soon or later potentially impact the society (Tip 10).

## Tip 10: Make your dataset, software code, and article open and publicly available

Once you carefully cleaned your dataset and engineered its features following the previous tips, it is a good idea to share it publicly. Make available all the original data and the associate metadata together with the processed data, metadata, and the software you developed for this purpose. If you have the authorizations to do so, or if the dataset was already released with an open, permissive license (such as the Creative Commons Attribution 4.0 International license, for example), we recommend to publicly share it online on repositories such as Kaggle [205], the University of California Machine Learning Repository [206], Zenodo [207], or FigShare [208].

Before releasing your dataset, we recommend to change the names of its features to something more meaningful and informative, if necessary. For example, in the past we worked with datasets where one of the variables was called “gender,” while its real meaning was “sex,” where 0 meant “male” and 1 meant “female”: we decided to change the name of that feature into “sex 0male 1female” [59,209]. We suggest to the readership to follow the same approach and to replace the names of the variables this way, to improve their clarity and informativeness.

Moreover, making your dataset publicly available will allow the reproducibility of your computational analysis and will bring new opportunities for secondary studies for any researcher worldwide [210]. For example, data of EHRs of patients with neuroblastoma released freely and openly on an open data repository can allow other scientists to perform secondary analyses and discover new aspects of this disease, which could eventually improve patients’ lives [211].

For the same reasons, we also recommend you to run your computational analyses with open source programming languages such as R or Python, and to publish your software code online on platforms such as GitHub, GitLab, or SourceForge [212,213]. Regarding the publication of the scientific articles of your studies, we suggest to submit your manuscripts to open access journals, so that once published, they will be publicly readable by anyone worldwide. Open access publications have a stronger impact and collect more citations [214]. A list of relevant open access journals on bioinformatics and medical informatics can be read on the Scimago Journal Rankings website [215,216]. Releasing articles as preprints on preprint servers such as bioRxiv, medRxiv, and arXiv can be another effective way to promote open scientific research.

## Tip 11: Always version and document your software and scripts

Similar to what is suggested for machine learning [217] and pathway enrichment analysis [218], we recommend to document all the steps of the data cleaning and feature engineering phases [219,220]. Version the produced data and metadata, and the software you developed for this purpose. Record both why you decided to do each step and the scientific and technical reasons of that decision in notebook [221], and insert comments in your software code explaining necessary information to understand what your scripts do. Write down parameters’ values and settings, too. As Sandve and colleagues [222] wrote: “For every result, keep track of how it was produced.” We completely agree. Moreover, it is important that you save and version-control all your data cleaning and feature engineering software scripts through tools such as Git, Apache Subversion (SVN), or Mercurial [223].

Having all the documentation up-to-date will be invaluable for anyone in the future who will need to understand your preprocessing steps, to explain some decisions you took to colleagues and collaborators, and possibly to explain your approach in a scientific article. Saving your scripts will be fundamental because you or your collaborators will likely need to repeat your preprocessing steps at some point. Well-written documentation can also become published tutorials, which can be useful to other users and researchers worldwide. An example of well-documented bioinformatics tutorial is the one on RNA-seq data workflow by Love and colleagues [224,225].

## Conclusion

Computational analysis of data is a key component of most scientific studies nowadays, in any scientific field. Although statistics, cluster analysis, and machine learning often play an important role in these projects, the quality of the results are influenced by steps performed on the original dataset before the actual computational analysis, such as data cleaning and feature engineering. These two preprocessing phases, although fundamental, sometimes are done poorly or in the wrong way by beginners and unexperienced researchers. In this context, we propose these quick tips for designing and performing data cleaning and feature engineering correctly and efficiently, in any scientific field.

Some of our tips relate to problems that are immediately visible to the researchers, such as how to handle missing data, while some other tips regard issues that would be unnoticeable to unexperienced users, such as how to handle technology noise. All in all, we believe our quick recommendations can serve as guidelines for researchers willing perform better computational analyses which can generate more robust and reliable results.
